# p53 and Ceramide as Collaborators in the Stress Response

**DOI:** 10.3390/ijms14034982

**Published:** 2013-03-01

**Authors:** Rouba Hage-Sleiman, Maria O. Esmerian, Hadile Kobeissy, Ghassan Dbaibo

**Affiliations:** 1 Department of Pediatrics and Adolescent Medicine, Division of Pediatric Infectious Diseases, Faculty of Medicine, American University of Beirut, P.O. Box 11-0236 Riad El Solh, 1107 2020 Beirut, Lebanon; E-Mails: me61@aub.edu.lb (M.O.E.); gdbaibo@aub.edu.lb (G.D.); 2 Department of Biochemistry and Molecular Genetics, Faculty of Medicine, American University of Beirut, P.O. Box 11-0236 Riad El Solh, 1107 2020 Beirut, Lebanon; E-Mail: hok03@aub.edu.lb

**Keywords:** ceramide, p53, apoptosis, sphingolipids, mitochondria, signaling, Bcl2 family, caspase

## Abstract

The sphingolipid ceramide mediates various cellular processes in response to several extracellular stimuli. Some genotoxic stresses are able to induce p53-dependent ceramide accumulation leading to cell death. However, in other cases, in the absence of the tumor suppressor protein p53, apoptosis proceeds partly due to the activity of this “tumor suppressor lipid”, ceramide. In the current review, we describe ceramide and its roles in signaling pathways such as cell cycle arrest, hypoxia, hyperoxia, cell death, and cancer. In a specific manner, we are elaborating on the role of ceramide in mitochondrial apoptotic cell death signaling. Furthermore, after highlighting the role and mechanism of action of p53 in apoptosis, we review the association of ceramide and p53 with respect to apoptosis. Strikingly, the hypothesis for a direct interaction between ceramide and p53 is less favored. Recent data suggest that ceramide can act either upstream or downstream of p53 protein through posttranscriptional regulation or through many potential mediators, respectively.

## 1. Introduction

Ceramide is a key sphingolipid that acts as a second messenger for multiple extracellular stimuli to mediate many cellular processes. Ceramide signaling, conserved throughout evolution, was found to be involved in death signaling in many systems. Since yeast cells undergo a cell death mechanism that resembles apoptosis, the sphingomyelin pathway appears evolutionarily older than the caspase-mediated death programs described in higher organisms [1]. Most DNA damaging agents and genotoxic stressors induce apoptosis in p53-dependent pathways. However, in the absence of p53, programmed cell death proceeds and is partly mediated by the “tumor suppressor lipid”, ceramide. Nevertheless, many stimuli can cause p53-dependent ceramide accumulation leading to cell death. In this review, we intend to focus on the role of ceramide in signaling pathways of apoptosis and try to shed light on its relation with p53.

## 2. Ceramide Biosynthesis

**Figure 1:**
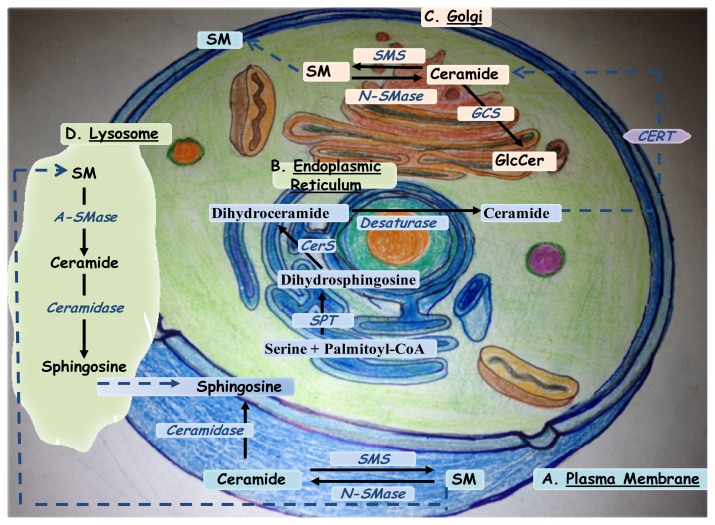
Metabolic pathways of ceramide synthesis and degradation: Names of organelles (A to D) are underlined. Names of enzymes are written in italic. Black solid arrows are used to show metabolic conversions. Blue dashed arrows indicate protein-mediated transfers. Abbreviations: SPT: Serine Palmitoyltransferase; CerS: Ceramide synthase; CERT: ceramide transfer protein. SMS: sphingomyelin synthase; A-SMase: Acid Sphingomyelinase, N-SMase: Neutral sphingomyelinase; GCS: Glucosylceramide synthase.

Ceramide is an N-acylsphingosine consisting of a fatty acid bound to the amino group of the sphingoid base, sphingosine. In general, ceramides are usually found with mono-unsaturated or saturated fatty acids of various lengths that significantly alter their physical properties. Many natural ceramides are being isolated and might be of therapeutic importance such as cameroonemide A from the plant *Helichrysum cameroonense*[2] and ceramide/cerebroside from the stem bark of *Ficus mucuso*[3]. Ceramides with 16–24 carbon fatty acyl chains are the most commonly found in mammalian cellular membranes. Depending on the cell type and stimulus, ceramide is generated by three major pathways (Figure 1). First, in the cell membrane, sphingomyelin can be broken down to ceramide in a reaction catalyzed by sphingomyelinases (neutral, acidic, or alkaline). Second, the *de novo* synthesis of ceramide occurs by the condensation of palmitate and serine to form 3-keto-dihydrosphingosine that is further reduced to dihydrosphingosine. This pathway, generating ceramide from less complex molecules, is catalyzed by the enzyme serine palmitoyl transferase (SPT) and occurs in the endoplasmic reticulum (ER). Dihydrosphingosine is then acylated by the enzyme (dihydro) ceramide synthase (CerS) of which there are 6 isoforms (CerS1-6) to produce dihydroceramide [4]. In its turn, dihydroceramide is then converted to ceramide by the dihydroceramide desaturase enzyme and transported to the Golgi by either vesicular trafficking or by the ceramide transfer protein CERT [5]. Endoplasmic reticulum–trans-Golgi membrane contacts are required for nonvesicular ceramide transport. These contact sites facilitate the transfer of newly synthesized ceramide from ER to sphingomyelin synthase (SMS) located at the trans-Golgi via CERT [5].

The third pathway is termed the salvage pathway. It contributes from 50% to 90% of sphingolipid biosynthesis, and occurs through the breakdown of complex sphingolipids and glycosphingolipids in acidic cellular compartments such as the late endosomes and lysosomes, to produce sphingosine. For instance, sphingomyelin can be converted to ceramide by acid sphingomyelinase, encoded by a distinct gene than that of neutral sphingomyelinase [6]. Furthermore, ceramide can be hydrolyzed by acid ceramidase to form sphingosine and a free fatty acid, both of which, and unlike ceramide, are able to leave the lysosome. Ceramide synthase family members probably trap free sphingosine released from the lysosome at the surface of the endoplasmic reticulum or in its associated membranes [3,4].

Additional studies revealed that variation in free Mg^2+^ causes sustained changes in membrane phospholipids and second messengers resulting in the activation of intracellular signal transcription molecules such as NF-κB, proto-oncogenes c-fos and c-jun, MAPK and MAPKK in vascular smooth muscle cells *in vitro*[7]. More importantly, variations in Mg^2+^ cause truncation of membrane fatty acids, significant activation of sphingomyelinase (SMase) and alterations in membrane sphingomyelin leading to the release of ceramides. Consequently, because of all these modifications, apoptotic caspases become activated and mitochondrial cytochrome c is released [8–10]. Furthermore, and contrary to sphingomyelinase, SMS directly regulates cellular ceramide and diacylglycerol (DAG) levels [11]. It was recently shown that Mg^2+^ deficiency upregulates SMS and p53 in diverse cardiovascular tissues and cells. Mg^2+^-deficient environments drive the *de novo* synthesis of ceramide via the activation of three enzymes in the sphingolipid pathway: SPT, SMS, and CerS. The lower the Mg^2+^ is, the greater is the synthesis of ceramide [12].

Although the cytoplasmic generated ceramide was described to play important roles in mediating signaling pathways, membrane ceramide share equivalent importance in mediating cellular pathways and functional processes. For instance, ceramide generated at the exoplasmic leaflet of the plasma membrane self-associates and mediates the formation of ceramide-rich platforms (CRPs) with diameters of 200 nm up to several microns. These macrodomains are thought to derive from sphingolipid and cholesterol-enriched rafts and seem to be active sites for protein oligomerization during transmembrane signaling [13]. However, some exceptions exist where membrane ceramide does not participate in signaling. For instance, in the breast cancer cell line MCF7, ceramide generation at the outer leaflet of the plasma membrane following the exogenous addition of bacterial sphingomyelinase does not induce cell death [14–16].

## 3. Ceramide and Cellular Signaling

Ceramide accumulates under specific conditions to play an important role in signaling pathways. Indeed, ceramide is a topological cell-signaling lipid that forms functionally distinct endomembrane structures and vesicles termed “sphingosome” that organize into a specialized apical compartment in polarized cells [17]. In general, growth factors, chemical agents, and environmental stresses generate ceramide in order to mediate proliferation, membrane receptor functions, immune inflammatory responses, differentiation, cell adhesion, growth arrest, or apoptosis [6,12,18–20]. Furthermore, there is evidence that ceramide mediates another terminal cellular event, senescence [21]. Indeed, ceramide contributes to senescence by activating the growth suppressor pathway through retinoblastoma (Rb) dephosphorylation and the mitogenic pathway mediated by c-Fos and AP-1 [22]. Moreover, ceramide can regulate other cellular mechanisms such as phagocytosis and autophagy. First, permeable C(6)-ceramide increases the cellular levels of endogenous ceramides via a sphingosine-recycling pathway leading to enhanced phagocytosis by Kupffer cell [23]. Second, MCF7 cells deficient in autophagy protein that were sensitive to photodynamic therapy presented an increase in ceramide levels [24]. In some cases, inhibiting the ceramide apoptotic pathway may lead to autophagy. For example, prostate cancer cell lines overexpressing acid ceramidase (AC) are resistant to ceramide-induced apoptosis because of the conversion of ceramide to sphingosine and consequently to the antiapoptotic sphingosine 1-phosphate. These cells were also found to have increased lysosomal density and increased levels of autophagy [25].

In addition to all the previously described roles, ceramide is involved in vesicular transport systems. Under normal physiological conditions, the binding of transferrin to its receptor generates ceramide at the cell surface through the activation of acid sphingomyelinase [26]. Ceramide self-assembles into domains that laterally sort transferrin receptors to clathrin-coated pits for endocytosis [26]. It was shown that in the absence of ceramide, lipid rafts take over to complete some mechanims. Under abnormal conditions where ceramide cannot be generated, transferrin/transferrin receptor complex translocates to the lipid rafts of the plasma membrane where it internalizes by clathrin-independent pathway [26]. Furthermore, in a recent study by Castro *et al.*, it was shown that ligand-bound Fas receptors oligomerize in lipid rafts independent of ceramide [27].

### 3.1. Ceramide and Hypoxia/Hyperoxia

Both hypoxia and hyperoxia are common stressors to which human and animal cells are exposed in the course of diseases and their treatment. Many studies were conducted to determine the effects of hypoxia or hyperoxia on ceramide. First, it was shown that upon exposure to chronic hypoxia, the myocardial mass was increased in a rat and mouse models of cyanotic congenital heart disease due to compensatory cardiac proliferation in the right ventricle (RV) [28,29]. This phenotype was associated with the absence of apoptosis, the relative decrease in total ceramide, specifically N-palmitoyl-D-erythro-sphingosine (C16-Cer) levels [30], and the significant increase of the precursor dihydro-N-palmitoyl-D-erythro-sphinganine (DHC16) in the RV. These findings suggested that dihydroceramide, and not only ceramide, plays a role in the RV adaptive response to hypoxia manifested by the survival of rat and mouse cardiomyocytes [29]. Studies in cultured cells exposed to hypoxia yielded different results compared to animal models. In a recent study done on H-SY5Y neuroblastoma cells, it was shown that hypoxia increased the ceramide concentration through *de novo* pathway and subsequently apoptosis was induced, with DNA fragmentation and ADP-ribose polymerase (PARP) cleavage [31]. In another study, the effect of hyperoxia was investigated on neonatal rat lung. It was shown that exposure to short term hyperoxia (3 days) induced apoptosis despite the increase in Bcl-2 through *de novo* synthesis of ceramide and overexpression of Bax. After 7 days of hyperoxia, animals adapted and survived the high oxygen levels by returning ceramide to baseline levels and reducing Bax and Bcl-2. However, prolonged hyperoxia (14 days) resulted in acute lung injury and absence of apoptosis despite the rise observed in Bax and ceramide levels probably because of a concomitant increase in the expression of Bcl-2 [32].

### 3.2. Ceramide Accumulation and Cell Death

Apoptosis occurs through either an intrinsic mitochondrial or an extrinsic death receptor pathway [33]. In the mitochondrial pathway, mitochondria are targeted either directly or through transduction by proapoptotic members of the Bcl-2 family, such as Bax and Bak. The mitochondria then release the apoptogenic protein cytochrome c leading to caspase activation and apoptosis. In the death receptor pathway, following the ligand binding, the receptors (Fas and tumor necrosis factor TNF) located at the cellular membrane recruit adaptor proteins such as Fas-Associated Death Domain (FADD) that then recruit pro-caspases, e.g., procaspase 8, which become activated upon clustering to initiate a caspase cascade. A crosstalk between both pathways is mediated via Bid, which becomes cleaved by apical caspases to target the mitochondria, and probably other still unknown factors [34].

The involvement of ceramide in apoptotic pathways has been widely studied. Sphingomyelin (SM), an immediate precursor to ceramide, is an important participant in key signal transduction pathways [35]. SM is localized mostly in the outer leaflet of the plasma membrane. Additionally, since SM gets synthesized in the *cis* and medial Golgi apparatus, the signaling pool of SM resides also in the inner leaflet of the plasma membrane or on the cytoplasmic face of a subcellular fraction (such as Golgi or endosomes) [36]. Indeed, many signaling lipid-regulated enzymes and lipases are associated with the inner leaflet of the plasma membrane; these include the ceramide-activated protein kinase C zeta (PKCz) [37] and ceramide-activated protein phosphatase (CAPP), as well as phospholipases C and A2 [35]. The exogenous use of bacterial sphingomyelinase allowed the examination of the role of outer leaflet sphingomyelin in inducing apoptotic signaling. Under these conditions, it was found that the generated ceramide was not enough to induce apoptotic responses [27]. The outer leaflet plasma membrane ceramide is associated with inhibition of PKC-induced nuclear factor-κB (NF-κB) activation [38], and of platelet-derived growth factor-induced phosphatidyl 3-kinase activity [39]. Thus, ceramide action appears to be compartmentalized. Indeed, when ceramide was generated endogenously by targeting bacterial sphingomyelinase to the mitochondria in MCF7 cells, it led to apoptosis marked with the death substrate poly (ADP-ribosyl) polymerase (PARP) cleavage [14]. However, when it was targeted to other compartments of the cell, no apoptosis was observed [14]. In this case, bacterial sphingomyelinase acts on both the inner and outer mitochondrial membrane pool of SM and the ceramide generated in either mitochondrial membrane can flip-flop from one leaflet to the other [7]. Furthermore, mitochondrial ceramide generation induces intrinsic apoptosis mediated by cytochrome c release. However, all these apoptotic events can be prevented by overexpression of Bcl-2s [14].

Under the same concept of implication of ceramide in cell death, several recent studies have shown that the biosynthetic pathway for ceramide generation differs depending on the type of cell and stimulus. Furthermore, depending on other cellular conditions, the generated ceramide can play dual functions inducing apoptosis or resulting in prosurvival according to its context of expression and to the cell type. For example, ionizing radiation (IR) induces *de novo* synthesis of ceramide and triggers HeLa cell apoptosis by specifically activating ceramide synthase isoforms 5 and 6 that preferentially generate C16 ceramide [40]. In these same cells, ceramide synthase 2 plays a protective role through the generation of C24 ceramides [40]. In another study using human monocytes U937, C16 ceramide was also shown to play an apoptotic role. Apoptosis was triggered by incubating these cells with palmitate. This resulted in an increase in cellular C16 ceramide and sphingomyelin, a decrease in reduced glutathione, and increase in reactive oxygen species (ROS) [41]. C16 ceramide was also described as crucial to induce the mitochondrial-mediated apoptosis observed in the adipose triglyceride lipase (Atgl^−/−^) null macrophages [42]. Contrary to these two previous studies, in squamous cell carcinomas (HNSCCs), C16 ceramide generated by CerS6 plays prosurvival antiapoptotic roles via ATF6/CHOP in response to endoplasmic reticulum-stress-induced apoptosis [43]. Similarly, C18 ceramide *de novo* synthesized by ceramide synthase 1 mediates the protective apoptotic response in human head and neck cancer instead of C16 ceramides in response to ER-stress and chemotherapy [44]. It was further shown that CerS1 gets cleaved and translocated from the endoplasmic reticulum to the Golgi apparatus with the help of protein kinase C in order to generate these ceramides [45]. All these findings shed light on the significance of the species of ceramide that is generated mediating a different cellular response according to the cell and stimulus types respectively.

**Figure 2:**
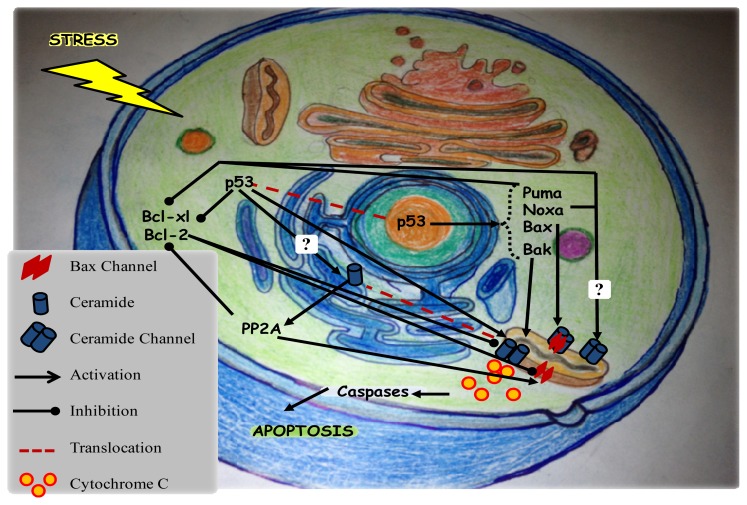
Mitochondrial apoptotic pathway involving p53 and ceramide in response to stress: Ceramide activates protein phosphatase 2A (PP2A) which dephosphorylates Bcl-2 and inhibits its antiapoptotic activity. Ceramide generated in the endoplasmic reticulum translocates to the mitochondria. Additionally, another pool of ceramide can be generated in the mitochondria. On the other hand, nuclear p53 activates the transcription of proapoptotic genes (Puma, Noxa, Bax and Bak). Bak elevates the activity of ceramide synthase in the mitochondrial outer membrane. Mitochondrial ceramides form large stable barrel-like channels either alone or with Bax. These channels are used to release cytochrome c to the cytoplasm resulting in activation of caspases and execution of apoptosis. Cytoplasmic p53 interacts with Bcl-xL in the mitochondria preventing it from disassembling ceramide and Bax channels. Puma and Noxa bind to antiapoptotic Bcl-2 family proteins freeing Bax and/or Bak from them. Noxa is also involved in the mitochondrial p53 and ceramide-dependent apoptosis but the specific pathway is still unclear.

In addition to *de novo* synthesis, ceramide can be generated by the sphingomyelinase pathways in response to apoptotic stimuli. This was shown in mitochondria of aged hearts where ceramide accumulated via the hydrolysis of sphingomyelin by neutral sphingomyelinase (N-SMase) [46]. In other cases, both pathways can be used simultaneously. For instance, in melanoma, interleukin-24 induces an ER stress triggered apoptotic response via the *de novo* synthesis of ceramide and acid sphingomyelinase (A-SMase) activity. In this context, activated protein phosphatase 2A (PP2A) was described to act downstream of the generated ceramide (Figure 2) contributing to apoptosis by dephosphorylation of antiapoptotic Bcl-2 and the Bcl-2 kinase PKCα [47].

Ceramide accumulation is often accompanied by ROS generation. In addition, ceramide can induce cell death through both caspase-dependent and caspase-independent mechanisms as described in mesenchymal stem cells derived from human adipose tissue (hASCs) [48]. Changes in endogenous levels of ceramide occur in the first stages of apoptosis such as activation of the initiator caspases, caspase 8, 9, and 10, and usually prior to the activation of the executioner caspases such as caspase 3, 6, and 7 [14]. Indeed, upon mitochondrial ceramide accumulation, cytosolic cytochrome c gets released from the mitochondria through ceramide channels [49]. In the cytosol, it forms a complex with Apaf-1 and procaspase 9, resulting in activation of caspase 9, which then activates other caspases (Figure 2), such as caspase 3, to control the execution of programmed cell death [14].

In a study on the cytotoxic effects of TNF-α, endogenous ceramide was generated by both hydrolysis of sphingomyelin and the *de novo* synthesis independently from TNF-α-induced activation of NF-κB [50,51]. During this apoptotic stress, ceramide trafficking for consequent sphingomyelin synthesis is reduced due to the disassembly of Golgi complex and the cleavage or inactivation of CERT by caspases 2, 3 and 9 [52]. In fact, cytokine response modifier A (CrmA), a potent inhibitor of some proteases, was found to inhibit ceramide generation and prevent TNF-α-induced death without affecting its ability to activate NF-κB [53]. All these results shed light on the existence of proteases that act upstream of ceramide formation in the signaling/activation phase of apoptosis. In this pathway, other mediators of the Bcl-2 family were identified to influence cell death mediated by ceramide. Among these are Bcl-2 and Bcl-xL which can protect from the apoptotic effects of TNF-α through different mechanisms of action. For instance, Bcl-xL acts on an upstream target of ceramide, whereas Bcl-2 functions on a downstream target of ceramide [54].

The involvement of ceramide in apoptosis has been studied in response to heat-shock in bovine oocytes [55]. In this context, ceramide was described as the key factor inducing germ cell apoptosis; the mechanism happens by translocating from cumulus cells into the adjacent oocytes and lipid rafts through gap junctions. Furthermore, ceramide has been shown to be involved in the mechanism of oocyte aging in a mitochondria-dependent mechanism. More specifically during aging, mitochondrial ceramide levels decrease and consequently alter the mitochondrial structure and function contributing to reduced oocyte quality [56]. Alternatively, short exposure to exogenous C8-ceramide was shown to strongly increase mitochondrial prohibitin (PHB) expression, maintaining mitochondrial integrity, and protecting germ cells. However, long exposure to C8-ceramide caused a decrease in PHB expression levels with consequent loss of mitochondrial cytochrome c, activation of caspases 3, and apoptosis [57]. Under the same concept, many studies focused on the effects of ceramide on mitochondria, key organelles mediating the intrinsic apoptotic response, in response to many apoptotic stimuli. For example, ceramide induces death of corneal stromal fibroblasts by HRK mediated mitochondria dysfunction. HRK, activator of apoptosis harakiri protein, translocates to mitochondria, where it interacts with mitochondrial protein p32 and BAD [58].

## 4. Mitochondrial Ceramide Channels

A key step in apoptosis is the release of pro-apoptotic proteins from mitochondria into the cytosol to initiate the execution phase. Ceramide influences the structure and function of mitochondria especially during early phases of apoptosis. Upon treatment of cells with C2-ceramide, mitochondria become fragmented, show reduced Ca^2+^ uptake, and collapsed membrane potential [14]. Mitochondrial ceramide has many sources. It can be exchanged from the endoplasmic reticulum in case the location of *de novo* synthesized ceramide on endoplasmic reticulum is close to the mitochondrial outer membrane (MOM) [59]. Alternatively, during apoptosis, the ceramide produced within the plasma membrane self-associates into platforms that subsequently invaginate and fuse with mitochondria. This stress-mediated plasmalemmal pool of ceramide is described to efficiently increase mitochondrial permeability [60]. Additionally, ceramide can be generated in the mitochondria by CerS1, CerS2, and CerS4 located at the outer mitochondrial membrane and by the reverse reaction of neutral ceramidase condensing sphingosine and fatty acyl-CoA [4,61,62]. Upon UV irradiation, acid sphingomyelinase migrates to mitochondria to help the mitochondrial ceramide synthase machinery in generating high level of ceramide [63–65]. Moreover, C16 ceramide can also be generated in the mitochondrial associated membranes via CerS5 and CerS6 [40].

Ceramide was described to self-assemble in the MOM to form large stable barrel-like channels (Figure 2) capable of releasing apoptotic proteins [49]. The permeabilized mitochondria probably contain only one or at most two channels of ceramide. The probability of new ceramide channel formation and the stability of existing channels depend on the steady-state concentration of ceramide in the membrane. In other words, permeabilization of mitochondria is controlled by the rates of ceramide synthesis, degradation, and transfer between membranes [66]. Methylation of either the amide nitrogen or the C1-hydroxyl group of ceramides disrupts the stability of channels and the mitochondrial permeabilization whereas, modifications of the C3 allylic hydroxyl group have no effect [67]. The structural features of ceramide are of great importance for the regulation of pro-apoptotic and anti-apoptotic Bcl-2 family proteins binding to ceramide channels. The C-4=C-5 trans-double bond has little influence on the ability of Bax and Bcl-xL to bind to the ceramide channels. Bax was found to interact with the amide group, the polar portion of the ceramide channel facing the bulk phase. However, Bcl-xL has an optimal interaction with long-chain ceramides that are elevated early in apoptosis and not short ones [68]. Based on this evidence of interaction, ceramide channels appear to be regulated by Bcl-2 family proteins. The anti-apoptotic proteins, Bcl-2 and Bcl-xL, were described to directly interact with ceramide channels (Figure 2) favoring their disassembly [66]. Bcl-xL was also described to inhibit ceramide accumulation in response to TNFα [51]. Bak and Bax are functionally redundant in the induction of apoptosis [69]. The activation of Bax depends upon the presence of Bak because the latter was described to elevate the activity of ceramide synthase in the mitochondrial outer membrane of mammalian cells in response to irradiation [70]. Consequently, ceramide forms a platform into which Bax inserts, oligomerizes and functions as a pore [71]. Indeed, there is an impact of ceramide on Bax monomers translocation to mitochondria and subsequent activation which is reflected by the localization of Bax oligomers in ceramide enriched microdomaines of mitochondria (Figure 2) called mitochondrial ceramide-rich macrodomains (MCRM) [71]. Therefore, both activated Bax and ceramide synergistically induce permeabilization of MOM during apoptosis [21]. Furthermore, it was shown that although activated Bax and ceramide directly interact to form a common mitochondrial permeabilizing channel, both ceramide and Bax can still be independent channel formers in membranes [72]. Bax can by itself or with the help of Bak form the mitochondrial permeabilization channel [73].

Although ceramide is described to trigger mitochondrial permeability, sometimes mitochondrial permeability transition pore (mPTP) opening can be differently regulated by certain ceramides. Although ceramide is known to mediate apoptosis, it has also been involved in cytoprotective processes like ROS-mediated preconditioning [74,75]. The proper mitochondrial function is strictly required in these ceramide mediated mechanisms. In fact, it was shown that upon deletion of the mitochondrial genome in *Saccharomyces cerevisiae*, C2-ceramide largely failed to cause ROS hypergeneration and cell death [76]. Moreover, ceramide regulates mPTP opening by temporally preventing it. It was shown that during ischemia in neuroblastoma cells, treatment with C2-ceramide leads to an increase in the formation of ROS, which induces a controlled protective opening of mPTP thus preventing mitochondrial Ca^2+^ overload [77]. However, ceramide synthase 6, localized to the inner mitochondrial membrane, generates C (16:0) ceramide upon an apoptotic stimulus. C (16:0) ceramide prevents mPTP opening, leading to increased Ca^2+^ accumulation in the mitochondrial matrix and a disturbance of mitochondrial Ca^2+^ homeostasis. The increase in mitochondrial Ca^2+^ activates calpain 10, a non-lysosomal mitochondrial enzyme that degrades the protein components of the pore resulting in its opening, rupture of the outer mitochondrial membrane and release of cytochrome c to initiate caspase activation. Interestingly, ceramide was found to react with oxidized cytochrome c and not reduced cytochrome c. Furthermore, glutathione known to reduce cytochrome c protects it from reacting with ceramide [78]. Depletion of intracellular glutathione with curcumin induces apoptosis in a ROS-independent manner, leading to caspase activation, inhibition of SMS activity, and induction of ceramide generation [79].

Ceramide can act both upstream and downstream of caspases. Upon irradiation, ceramide can be accumulated in response to Fas ligand treatment associated with apoptosis induction in Jurkat leukemia T cells. In this example, for ceramide to be generated and the mitochondrial apoptotic pathway to be efficiently activated, caspase 8 and 10 are essential. Consequently, accumulated ceramide enhances caspase 9 activation followed by caspase 3 activation and PARP cleavage [80].

## 5. Ceramide and Related Molecules

Ceramide along with some other sphingolipids are present in high levels in nervous tissues. A correlation between the acyl chain composition of these sphingolipids, ceramide and neurological diseases was established. More specifically, ceramide synthase expression and activity in the brain is linked to the different acyl chain compositions of ceramide and complex sphingolipids in a number of neurodegenerative diseases and conditions [81]. Although ceramide appears to be the major signaling sphingolipid, several ceramide metabolites have emerged to play an important role in various signaling pathways. In addition to ceramide, sphingosine 1-phosphate (S1P) and ceramide 1-phosphate (C1P) facilitate the activation of proinflammatory transcription factors to upregulate the proinflammatory cyclooxygenase-2 (COX2) and prostaglandins [82]. Sphingosine-1-phosphate mediates, with or without G-protein-coupled S1P receptor signaling, prosurvival, angiogenesis, metastasis and/or resistance to drug-induced apoptosis [83]. Indeed, S1P is named the “anti-apoptotic metabolite” of ceramide. It is generated by the phosphorylation of sphingosine by sphingosine kinase 1 (SK1), an enzyme that regulates various aspects of cell survival and proliferation and is itself regulated via proteases and p53 [84]. Ceramide 1-phosphate on the other hand, is important for membrane biology and for the regulation of membrane-bound proteins. Ceramide kinase (CERK), which catalyses the production of C1P, appears to be tightly regulated in order to control both ceramide levels and production of C1P. However, recent studies revealed alternative unknown C1P-producing mechanisms [85]. C1P has a dual regulatory capacity acting as an intracellular second messenger to regulate cell survival, or as extracellular receptor ligand to stimulate chemotaxis [86]. Indeed, C1P was found to induce the proliferation of primary bone marrow-derived macrophages through the formation of ROS by NADPH oxidase [87]. In another study, C1P was described to stimulate proliferation in macrophages by activation of the mammalian target of rapamycin (mTOR) [88] and in myoblasts by phosphorylation of glycogen synthase kinase-3 β, upregulation of cyclin D1, and activation of both phosphatidylinositol 3-kinase/Akt and ERK1/2 pathways [89]. Another signaling molecule related to ceramide is diacylglycerol (DAG). Since the translocation of lipids across membranes (flip-flop) is an important biological process, especially for lipids involved in cellular signaling, both ceramide and DAG translocate from one leaflet to another on the plasma membrane. Interestingly, they both have equal preference for both raft and nonraft membranes and display relatively small free energies of exchange which may have implications for their signaling and membrane localization [90]. At a functional level, DAG appears to play an opposite role to ceramide where it is known to activate PKC and inhibit apoptosis [91], whereas ceramide specifically inhibits PKCθ and α thereby promoting apoptosis [92].

Many other direct targets of ceramide have been identified including CAPK and CAPP [6,93]. An example of CAPK is c-Raf. Ceramide specifically binds to the kinase c-Raf and activates it, leading to the activation of the MAPK cascade [94]. PP2A is considered the best characterized CAPP. Ceramide can mediate PP2A upregulation and localization to the Golgi apparatus in HEK293 cells in response to microcystin-LR [95]. Furthermore, ceramide recruits PP2A to the mitochondria where it dephosphorylates Bcl-2 and causes its loss of antiapoptotic activity [96].

## 6. Ceramide in Yeast

Yeast cells were shown to have both glycosylphosphatidylinositol-anchored proteins and sphingolipids. Indeed, sphingolipids are important structural components of yeast membranes that play important roles such as protein trafficking, cell death regulation, and intracellular signaling through modulation of protein kinases or phosphatases [97]. Mature sphingolipids of *Saccharomyces cerevisiae* consist of inositolphosphorylceramides (IPCs) containing C26:0 or C24:0 fatty acids, mannosyl-IPCs (MIPCs), inositolphosphoryl-MIPCs (M(IP)2Cs), phytosphingosine and dihydrosphingosine [98,99]. In yeast cells, ceramide is synthesized in the endoplasmic reticulum by ceramide synthases Lag1p and Lac1p and transferred to the Golgi apparatus where inositolphosphorylceramide (IPC) is formed [100]. Since ceramide has been shown to be involved in the unfolded protein response (UPR) induction in yeast cells, as in mammalian cells, this may reflect the conservation of UPR response between yeast and mammals. Moreover, the enzyme responsible to generate ceramide in rat pancreatic INS-1E cells was described to be ceramide synthase 6 [101]. Similarly to mammalian cells, serine/threonine phosphatases have also been described in the cell cycle regulation of yeast cells. For instance, fission yeast ppe1 phosphatase was identified to play a role in cell morphogenesis and mitosis [102]. Interestingly, the budding yeast protein phosphatase Sit4p was shown to be the catalytic subunit of a ceramide-activated protein phosphatase in budding yeast [103].

Ceramides help transport GPI-anchored proteins to the Golgi apparatus [104,105]. Cells lacking all known ceramide synthases, are still capable of producing ceramides, which will be added after their synthesis, to GPI anchors and used for the synthesis of small amounts of normal IPCs essential for viability and longevity of yeast [99]. For instance, M(IP)2Cs regulate the toxicity of zymocin as a secondary membrane receptor required for γ-toxin uptake [106]. In addition, yeast plasma membrane H^+^-ATPase Pma1p is one of the most abundant proteins to traverse the secretory pathway. Its oligomerization depends on the presence of ceramide before its transport in COPII vesicles [107].

Ceramide is involved in cell death programs in yeast cells through either the addition of exogenous ceramide analogs or the induction of intracellular ceramide accumulation. For instance, cell-permeable short chain C2-ceramide induces the generation of reactive oxygen species (ROS) in rapid proliferating *S. Cerevisiae* cells [76]. C2-ceramide results in a dose-dependent inhibition of proliferation similarly to mammalian cells [108]. Upon exposure to heat stress, *S. cerevisiae* activates SPT and accumulates ceramide that subsequently induces a transient cell cycle arrest [18]. In addition, cell death in these yeast cells induced by ceramide causes ROS generation through the mitochondria. However, this cell death is caspase-independent non-apoptotic death [109]. These novel findings obtained in a simple unicellular organism describe ceramide as a central mediator in ancient cell death pathways [110]. Since caspase-independent cell death and ceramide signaling appear in yeasts cells, this indicates that ceramide is an ancient cellular response to stress that evolutionarily predates the appearance of caspases and apoptosis.

## 7. p53 and Apoptosis

p53 is the most studied tumor suppressor. Known as the guardian of the genome [107], its overexpression may modulate cell survival or death depending on its genetic profile. When up-regulated, p53 appears to preserve genome integrity by serving three essential functions. First, p53 plays an important role in the regulation of the cell cycle where its up-regulation results in the arrest of the cell in the G1 phase [111]. Second, p53 up-regulation can drive the cell towards apoptosis [112,113]. Third, p53 appears to be involved in DNA repair mechanisms [114–116].

In many pathological conditions such as cancer, neurodegeneration, ischemia, cholestasis or atherosclerosis, p53 is either found to be dysregulated, dysfunctional, inactivated, mutated or silent [117]. For instance, in cancer therapy, restoration of p53 function is very promising for achieving tumor regression [118]. In case of cervical carcinoma, which develops as a result of human papillomavirus (HPV) infection, p53 is degraded by the HPV E6 protein [119]. Other viral proteins also target p53 to dysregulate its function such as the adenoviral E1B55K [120], large T antigen of SV40, Tax of the Human T lymphotropic virus type I and EBNA5 of Epstein-Barr virus [121]. The CRPVE6 protein of cottontail rabbit papillomavirus (CRPV) interacts with the histone acetyltransferase p300 and inhibits the ability of p53 to induce apoptosis [122]. This targeting of p53 by viral proteins underscores the importance of p53 in the defense against the stress of viral infection. Interestingly, p53 is not alone to regulate the numerous cell functions such as cell cycle arrest, senescence and apoptosis. Following the discovery of p53, two p53-homologs, p63 and p73 were described to induce cell cycle arrest and apoptosis. They possess 60% homology with the p53 DNA binding domain that regulates p53 target genes [123].

### 7.1. Mechanism of Action

The relationship between p53 and apoptosis has been the focus of many researchers in many studies. Activation of p53 induces apoptosis typically through the mitochondrial pathway, although p53 can also modulate cell death through death receptors. A recent study was done to elucidate how p53 and the Bcl-2 protein family mediate apoptosis in pancreatic ductal adenocarcinoma [109]. The expression of antiapoptotic Bcl-2 and proapoptotic Bcl-xS was significantly associated with p53; furthermore, Bcl-2/Bax ratio was significantly associated with apoptosis [124].

p53 activates the transcription of various proapoptotic genes, including those encoding members of the Bcl-2 family (Figure 2), such as the BH-3 only proteins Bax [125], Noxa, and p53-upregulated modulator of apoptosis (Puma) [126]. This transcriptional activity is sequence specific and relies mainly on the DNA-binding domain encoded by the sequence from exon 4 to exon 8 of p53 [118]. p53 was found to be involved in a novel proapoptotic pathway contributing to the progression of heart failure through the transcriptional activation of Puma, a BH3-only member of the Bcl-2 family [127]. Alternatively, p53 can also trigger apoptosis by repression of antiapoptotic genes, such as Bcl-2 [128,129] and survivin, thus promoting caspase activation [130]. Overexpression of p53 stimulates Fas transcription in the spleen, thymus, kidney, and lung [131] and enhances cell surface levels of Fas by promoting its transport from Golgi complex [132]. In response to DNA damage, p53 along with NF-κB, activates DR5, the death domain-containing receptor for TNF-related apoptosis-inducing ligand (TRAIL) and promotes cell death through caspase 8 [133,134]. Pro-apoptotic activation of Bid, a nuclear-cytoplasmic protein, may be negatively regulated by its phosphorylation in response to DNA damage. Bid is transcriptionally regulated by p53, and both can be exported from nucleus to the mitochondria in response to DNA damage [135].

Noteworthy, although nuclear p53 can induce autophagy through transcriptional effects, cytoplasmic p53 acts as a master repressor of autophagy [136,137]. In fact, p53 plays a transcription-independent proapoptotic role in the cytoplasm and/or mitochondria in addition to its nuclear transcriptional role. The targeting of p53 to the mitochondria induces a rapid apoptotic response as efficiently as the transcription-dependent response. The protein Tid1, cochaperone of the heat shock 70 protein, directly interacts with p53, resulting in mitochondrial translocation of the complex and induction of intrinsic apoptosis under hypoxic or genotoxic stresses [138]. In addition, physical and functional interactions of p53 with various members of the Bcl-2 family provide the basis for the transcription-independent apoptotic route [139–141]. In response to stress, a pool of stabilized cytoplasmic p53 proteins consisting of unubiquitinated or monoubiquitinated p53 translocate to the mitochondria [34]. This was described to be involved in Bax translocation to mitochondria in simvastatin-induced apoptosis [142]. The cytosolic p53 induces homo-oligomerization of Bax (Figure 2), followed by Bax activation and mitochondrial translocation [143]. In mitochondria, p53 physically interacts with antiapoptotic Bcl-xL and Bcl-2 and antagonizes their protective antiapoptotic effects. Although p53 performs its main role in the mitochondrial outer membrane, through the p53/Bcl-xL specific pathway, it also interacts with different proteins in the mitochondrial inner membrane and matrix involved in different apoptotic mechanisms [144]. In response to stress conditions, nuclear p53 induces transcription of *puma*. Puma in its turn activates cytoplasmic p53 by dissociating it from Bcl-xL in the mitochondria as well as frees Bax and/or Bak from antiapoptotic Bcl-2 family members [145]. In the absence of functional p53 and Bax, apoptosis becomes mediated by the tumor suppressor p14 (ARF) through Bak activation [146]. Additionally, activating Bak is facilitated by down-regulating anti-apoptotic Mcl-1 and Bcl-xL which allows mitochondrial permeability shift, release of cytochrome c, activation of caspases, and subsequent fragmentation of genomic DNA [146]. A recent study suggested that caspase 9 and its adaptor Apaf-1 may be involved in mediating the effects of p53 on apoptosis [147].

### 7.2. Regulation of p53

p53 function is regulated by kinases and phosphatases. It is serine-phosphorylated by many stress activated kinases such as cyclin dependent kinase 5 (Cdk5) [148]. Activation of p53 under non stress conditions is poorly understood. Therefore, the detection of upstream kinases that phosphorylate non-genotoxically overexpressed p53 is of a promising therapeutic impact on cancer. A recent study by Ajay *et al.* showed that inhibition of protein phosphatase 2A (PP2A) activates p53 [149]. Furthermore, overexpressed p53 gets activated by getting phosphorylated at Serine20 and Serine46 residues by cyclin dependent kinase 5 [149]. Consequently, p53 gets recruited to p21 and Bax promoters that respectively induce G2 arrest and apoptosis through intrinsic mitochondrial pathway [149]. HIPK2, a stress-induced kinase, functionally cooperates with p53 to suppress cancer. It participates in the Serine46 phosphorylation and Lysine382 acetylation of p53. HIPK2, with the help of SIRT1 deacetylase, can also downregulate Nox1, an inhibitor of p53 Lysine382 acetylation [150].

p53 is negatively regulated by Wip1, a stress-response phosphatase that presents an attractive target in the treatment of many tumors. It was shown that overexpression of Wip1 increased anticancer drug sensitivity of p53-null tumors. The increased sensitivity resulted from activation of the intrinsic pathway of apoptosis through increased levels of the pro-apoptotic protein Bax and decreased levels of the anti-apoptotic protein Bcl-xL [151].

Early during apoptosis, golgi-vesicle-tethering protein p115 is cleaved by caspases and its 205 amino acid C-terminal fragment (CTF) translocates to the nucleus. This nuclear localization regulated by SUMOylation is responsible for inducing apoptosis. It was shown that expression of the CTF leads to the phosphorylation and stabilization of p53 and results in the expression of Puma. CTF expression also promotes p53-ERK interaction, which amplifies the apoptotic signal [152].

In addition to phosphorylation, p53 can be regulated by several other modifications. Glycogen synthase kinase-3 (GSK-3) is required for the S86 phosphorylation and activation of p53-acetyltransferase Tip60 as well as the induction of proapoptotic BH3-only protein Puma [153]. Acetylation of p53 by Tip60 at Lysine120 residues protects it from ubiquitination by its specific repressor mouse double minute-2 (Mdm-2) oncoprotein [154]. Mdm-2 protein can inhibit p53 by regulating its stability, cellular localization, and transactivation [155]. Methylation, sumoylation, and neddylation also regulate p53 protein stability and transcriptional activation of different subsets of target genes [156].

X chromosome-linked inhibitor of apoptosis protein (XIAP)-associated factor 1 (XAF1) interacts with p53 and regulates its role in inducing apoptosis in human gastric and colon cancer cells. Wild-type p53, but not mutant p53, down-regulates XAF1 at both mRNA and protein levels. A novel feedback loop was described between XAF1 and wild-type p53, whereby over-expression of XAF1 leads to activation of wild-type p53 via post-translational modification, resulting in p53 nuclear accumulation, increased transcriptional activity and enhanced p53-dependent apoptosis [157].

### 7.3. Apoptosis Independent of p53

While p53 is required to induce cell death upon DNA damage, p53-independent responses can be observed in cells that present inactive cell cycle checkpoints [158]. For instance, in cancer cells lacking p53 and cell cycle arrest signaling molecules ATM, ATR, Chk1, and p38 MAPK/MK2, caspase 3 and mitotic catastrophe become activated following DNA damage [159]. Moreover, in zebrafish embryos lacking functional p53 and Chk1, ATM and ATR are required to activate a caspase 2 apoptotic response, independent of caspase 9 or caspase 3 [160]. In colon inflammation or chronic inflammation of pancreatic cells, Puma gets activated by NF-κB and endoplasmic reticulum stress and then leads to an intrinsic apoptosis pathway independent of p53 and involving mitochondrial Bax translocation, cytochrome c release, and caspase 3 cleavage [161,162]. In a recent study, benzo[a]pyrene (B[a]P), a constituent of cigarette smoke was shown to generate genotoxins and ultimal carcinogen molecules that cause DNA mutations. In case of unrepaired damage, p53, Bax, p38, JNK and stress-activated protein kinases (SAPKs) get involved to stop proliferation and induce apoptosis. However, SAPK operates independently of p53 and controls apoptosis by a novel mechanism possibly downstream of caspases [163]. In addition to SAPK and cell cycle checkpoints, Bcl-2 family members acting downstream of p53 can induce apoptosis independently of p53. In gastric cancer cells, overexpression of Bak was found to induce apoptosis along with the activation of caspase 3 [164].

p53 gene is frequently mutated in human tumors, which contributes to chemotherapeutic resistance or poor responsiveness. The previous findings are of clinical importance for treatment of p53 mutant or resistant cancer cells whereby activation of alternative mediators can induce apoptosis independent of p53. Thus, a lot of research is being conducted to identify chemotherapeutic agents that act independently of the p53 pathway. For instance, paclitaxel is an anti-microtubule agent that stabilizes microtubules leading to mitotic arrest followed by apoptosis in both wild type p53 and defective-p53 tumors [165]. Nevertheless, p53 mutation status cannot be used as an exclusive indicator to predict the chemotherapy response of human cancer xenografts. Instead, the expression profile of p53-related proteins must also be taken into consideration [166].

## 8. Ceramide and p53

### 8.1. Ceramide and p53 in Apoptosis

Both ceramide and p53 have been shown to play important roles in mediating apoptosis (Figure 2). In many cases, apoptosis can be mediated independent of p53. However, several studies were conducted to elucidate the pathways involving both actors in the aim to identify possible interactions between them. Dbaibo *et al.* showed that exposure of Molt-4 leukemia cells to chemotherapeutic agents such as actinomycin D or to γ-irradiation induces p53-dependent ceramide accumulation and cell death [167]. Upon p53 up-regulation, ceramide is generated through *de novo* ceramide synthesis, specifically associated with ceramide synthase 5 activities rather than SPT activity [168]. However, ceramide did not up-regulate p53 expression and p53 was not required for ceramide-induced effects as cells lacking p53 died equally in response to exogenous ceramide. Taken together, these data suggested that p53 operates upstream of ceramide accumulation in p53-dependent pathways [143]. In other studies, the chemotherapeutic agent, daunorubicin, functions in a p53-dependent manner by elevating ceramide through activation of ceramide synthase [169] or through neutral sphingomyelinase [170]. Thus, it appears that the p53-regulated stress response evolved at a later stage compared to ceramide and that p53 engages the ceramide-regulated stress response to exert some of its biological functions. Thus, multiple mechanisms of elevation of endogenous ceramide after p53 up-regulation are possible and this may be dependent on cell and stimulus type.

Additional studies were done on other models such as *Caenorhabditis elegans*. The irradiation of *C. elegans* germ cells induced mitochondrial apoptotic death via both p53 and ceramide following double-strand breaks in the DNA [171]. In this model, both ceramide synthesis via ceramide synthase and CEP-1/p53-EGL-1 (BH3-only protein) pathway via DNA damage–activated cell cycle checkpoint genes are responsible to trigger caspase activation and apoptosis [172]. Likewise, several other reports confirmed that ceramide accumulation is an important downstream mediator of the p53 response [173,174].

Studies are ongoing to better understand the detailed pathways linking ceramide and p53 in apoptosis. The late apoptotic stage involving executioner caspases and PARP cleavage is common between p53-dependent and independent ceramide accumulation. However the detailed early apoptotic signals involving both p53 and ceramide, such as the mediators of the p53-dependent ceramide accumulation in apoptosis, remain unknown. The hypothesis for a direct interaction between ceramide and p53 is less favored but indirect interactions are more likely. In a recent study, suppression of glucosylceramide synthase was described to restore p53-dependent apoptosis in p53-mutant cancer cells through ceramide. In fact, data suggested that ceramide restored the wild-type p53 expression at posttranscriptional processing in the latter study [175]. Because glucosylceramide synthase catalyzes ceramide glycosylation, converting ceramide to glucosylceramide, its silencing increases ceramide and decreases glucosylceramide and other glycosphingolipids. Although it is still not clear how ceramide modulates p53 resuscitation, several studies suggest that it mediates posttranscriptional modifications as it does for caspase 9 and Bcl-xL in cancer cells [176,177].

Based on these previous studies, the relation between ceramide and p53 with respect to apoptosis remains controversial and conditional. Additionally, the correlation between p53 and ceramide was described in senescence. Exogenous ceramide was shown to act upstream of p53, such as in Ras-induced senescence [178]. For instance, exogenous C2-ceramide treatments in primary cortical neuron cultures, mouse B cell lymphoma cell line, and mouse fibroblasts were found to increase cellular p53 [179]. Since the upregulation of p53 upon exposure to ceramide is cell type dependent, many further studies are needed in order to clarify this relationship.

### 8.2. Ceramide and p53 in Cell Cycle Arrest

p53, guardian of the genome, tightly controls the cell cycle. Its up-regulation results in the arrest of the cell in the G1 phase [111]. The protein p21 (WAF1/Cip1) is a p53-inducible gene product that mediates some of its effects on cell cycle regulation [180,181]. It acts by inhibiting G1 cyclin dependent protein kinases (Cdks) which phosphorylate the Rb protein and related family members [182,183] leading to a G0/G1 arrest of the cell cycle [183]. Similarly, accumulation of endogenous ceramide or the exposure to exogenous C6-ceramide induces a G0/G1 arrest of the cell cycle in Molt-4 cells [184]. It was shown that ceramide mediates this effect via ceramide activated protein phosphatase 1 that dephosphorylates Rb protein [185]. Furthermore, exogenous ceramide was also found to influence cell cycle progression of MCF-7 cells by inducing an arrest in the G1 phase. This arrest is concomitant with a decreased expression of cyclins D and E and increased expression of p53 and p21. Interestingly, inhibition of p53 sensitized the MCF-7 cells to ceramide-induced cell death [186]. These studies shed light on the existence of a cross-talking between the ceramide mediated pathway and p53-mediated pathway with respect to the cell cycle arrest.

## 9. Ceramide and Cancer

Ceramide and p53 were shown to be concomitantly upregulated in response to various cell stressors [187,188]. Most of DNA damaging agents and genotoxic stresses induce apoptosis in p53-dependent pathways. However, in the absence of p53, programmed cell death can still be mediated by the proapototic ceramide. In p53-independent systems, such as growth suppression induced by TNF-α or serum deprivation, ceramide can still accumulate and signal for apoptosis, irrespective of p53 status [167,189,190]. Since most cancers become either p53-mutant or p53-defective following initial treatment, the identification of alternative therapeutic targets or tumor suppressors that can activate apoptosis independently of p53 becomes crucial. Indeed, ceramide proved to act downstream of p53 and therefore can be used in these types of cancers to activate apoptosis. Therefore, the manipulation of endogenous ceramide by inhibiting enzymes that metabolize it such as ceramidases and glucosyltransferases is of great importance when coupled to cancer treatments. In fact, p53-deficient osteosarcoma and colon cancer cells were sensitized to the mitomycin C treatment when coupled to ceramide glucosyltransferase inhibition [191]. In recent studies, sphingomyelinases and ceramide synthases were identified as important targets for γ-irradiation and chemotherapeutic drugs in the treatment of cancers as many of these treatments induce cell death via the generation of ceramide. Therefore, manipulation of ceramide production and metabolism is a promising tool for the enhancement of anti-tumor therapies [192]. Complex dietary sphingomyelin and glycosphingolipids were found to inhibit the development of colon cancer through a protective role played by their bioactive metabolites ceramide, sphingosine, and sphinganine. These latter sphingolipids were described to inhibit proliferation and stimulate apoptosis in the human colon cancer cells [193]. Additional sphingolipid regulating enzymes have also been implicated in the accumulation of ceramide in cancer cells. It was shown that the irradiation of human breast cancer cell line T47D increased the activities of β-glucosidase, β-galactosidase, sialidase, and sphingomyelinase up to 72 h. These enzymes are used to breakdown more complex sphingolipids into ceramide. After irradiation, plasma membrane ceramide was increased, cell proliferation reduced and apoptotic cell death increased [194]. In a study by Camgoz *et al.*, a tyrosine kinase inhibitor, nilotinib, was found to induce apoptosis in human chronic myeloid leukemia (CML) cells through upregulation of ceramide synthase genes and downregulation SK1 [195]. A recent study has shown that tamoxifen, an inhibitor of ceramide glycosylation, increases the apoptotic efficiency of C6-ceramide by blocking its anabolism and maintaining its availability [196]. Acid ceramidase is considered a central player in ceramide metabolism by catalyzing the hydrolysis of pro-apoptotic ceramide to sphingosine, which can then be converted to anti-apoptotic S1P by SK1 [197]. Since acid ceramidase is found to be upregulated in prostate cancer and in some breast tumors, it is considered a putative anticancer target and its inhibition sensitizes breast cancer cells to C6-ceramide treatments [198].

The mechanisms of action of ceramide in cancer vary from one cancer type to another but include the mechanisms of induction of apoptosis. Additional mechanisms have also been described. First, the role of ceramide in enhancing apoptosis in some cancer cells can be mediated by the inactivation of Akt/mTOR pathway through the activation and release of protein phosphatase 1 (PP1), which then dephosphorylates AKT and causes cancer cell death [199]. In three acute myeloid leukemic cell lines (HL-60, NB4 and U937), different mediators were shown to act with ceramide to induce apoptosis in response to fenretinide treatment [200]. In HL-60 cells, ROS function upstream of ceramide to induce apoptosis unlike NB4 and U937 cells where apoptosis requires ROS independently from ceramide [200]. Furthermore, using a hepatocellular carcinoma cell line Huh7, selenite treatment combined with SK1 inhibitor was found to sensitize the cells to the cytotoxic effects of selenite through ROS generation and ceramide accumulation [201]. In addition, C8-ceramide and SK-1 inhibitor synergistically potentiate the cytotoxic and apoptotic effect of resveratrol, by increasing ceramide levels in chronic myelogenous leukemia cells [202]. All these observations point towards an important role for ceramide in the induction of apoptosis in cancer cells.

## 10. Conclusions

Ceramide and p53 collaborate to mediate apoptosis in response to various cellular stresses. Many signaling pathways involving this “tumor suppressor” couple are still unclear. Based on numerous studies, it is more probable that ceramide operates downstream of p53 in mediating apoptosis. Additionally, cell death mediated by ceramide in yeast cells is caspase-independent which suggests that ceramide is an ancient cellular process that evolutionary precedes the appearance of caspases and apoptosis. Further studies are required in order to define the pathways of p53-dependent and independent responses; this will allow a better identification of the events downstream from ceramide generation. These events are expected to vary depending on the type of cell and cytotoxic stress. Any future contribution to the research area of “ceramide pathways” might identify promising therapeutic targets in chemotherapy of p53-deficient cancers. Consequently, ceramide and sphingolipids can become more involved in pharmaceutical/pre-clinical research and later in clinical trials of cancer cases.
